# Potential Nutraceutical Properties of Leaves from Several Commonly Cultivated Plants

**DOI:** 10.3390/biom10111556

**Published:** 2020-11-15

**Authors:** Hafsa Amat-ur-Rasool, Fenella Symes, David Tooth, Larissa-Nele Schaffert, Ekramy Elmorsy, Mehboob Ahmed, Shahida Hasnain, Wayne G. Carter

**Affiliations:** 1 School of Medicine, University of Nottingham, Royal Derby Hospital Centre, Derby DE22 3DT, UK; hafsa.phd.mmg@pu.edu.pk (H.A.-u.-R.); mzyls6@exmail.nottingham.ac.uk (L.-N.S.); ekramyelmorsy@mans.edu.eg (E.E.); 2 Department of Microbiology and Molecular Genetics, University of the Punjab, Lahore 54590, Pakistan; mehboob.mmg@pu.edu.pk (M.A.); Shahida.mmg@pu.edu.pk (S.H.); 3 School of Pharmacy, University of Nottingham, Nottingham NG7 2UH, UK; fenellasymes@googlemail.com; 4 School of Life Sciences, University of Nottingham, Nottingham NG7 2UH, UK; David.tooth@nottingham.ac.uk; 5 Department of Forensic Medicine and Clinical Toxicology, Faculty of Medicine, Mansoura University, Mansoura 35516, Egypt

**Keywords:** Alzheimer disease, acetylcholinesterase inhibitors, antioxidants, butyrylcholinesterase inhibitors, molecular modelling, nutraceuticals, phytochemicals

## Abstract

Chronic dietary ingestion of suitable phytochemicals may assist with limiting or negating neurodegenerative decline. Current therapeutics used to treat Alzheimer disease elicit broad adverse drug reactions, and alternative sources of cholinesterase inhibitors (ChEIs) are required. Herein, we screened methanolic extracts from seven commonly cultivated plants for their nutraceutical potential; ability to inhibit acetylcholinesterase (AChE) and butyryl-cholinesterase (BuChE), and provision of antioxidant activity through their 2,2-diphenyl-1-picryl-hydrazyl-hydrate (DPPH) free radical scavenging capabilities. Potential neurotoxicity of plant extracts was examined via application to SHSY-5Y neuroblastoma cells and quantitation of cell viability. Methanolic extracts of *Citrus limon* (Lemon), *Bombax ceiba* (Red silk-cotton), *Lawsonia inermis* (Henna), *Eucalyptus globulus* (Eucalyptus), *Ocimum basilicum* (Basil), *Citrus reticulata* (Mandarin orange), and *Mentha spicata* (Spearmint) all displayed concentration-dependent inhibition of AChE and BuChE. The majority of extracts inhibited AChE and BuChE to near equipotency, with Henna and Eucalyptus extracts the two most potent ChEIs. All plant extracts were able to scavenge free radicals in a concentration-dependent manner, with Eucalyptus the most potent antioxidant. Toxicity of plant extracts to neuronal cells was concentration dependent, with Eucalyptus also the most toxic extract. Fractionation of plant extracts and analysis by mass spectrometry identified a number of plant polyphenols that might have contributed to the cholinesterase inhibition: 3-caffeoylquinic acid, methyl 4-caffeoylquinate, kaempferol-acetyl-glycoside, quercetin 3-rutinoside, quercetin-acetyl-glycoside, kaempferol 3-*O*-glucoside, and quercetin 3-*O*-glucoside. In silico molecular modeling of these polyphenols demonstrated their improved AChE and BuChE binding affinities compared to the current FDA-approved dual ChEI, galantamine. Collectively, all the plant extracts contained nutraceutical agents as antioxidants and ChEIs and, therefore, their chronic consumption may prove beneficial to combat the pathological deficits that accrue in Alzheimer disease.

## 1. Introduction

Globally, we are living longer and with a demographic shift such that the proportion of the population over 60 has grown dramatically, estimated to be approximately 1 billion in 2017 but expected to double by 2050 [1]. As a consequence of population growth, the number of people living with dementia doubled between 1990 and 2016 to approximately 44 million [2] and the current number of approximately 50 million sufferers is expected to triple by 2050 [3].

Age itself represents the greatest risk factor for dementia and associated disability-adjusted life years [4]. Hence, living longer does not guarantee living healthier.

Additionally, there are other risk factors for dementia and Alzheimer disease (AD) including genetic predispositions [5] and environmental exposures [6], as well as lifestyle factors including smoking [4], excessive consumption of alcohol [7], and diet, such as levels of dietary fats [8].

The benefits of diets rich in fruits and vegetables as well as a low consumption of alcohol have been purported to contribute to reduced dementia risk and the health and longevity associated with a ‘Mediterranean lifestyle’ [9,10,11,12,13,14]. One of the proposed mechanisms responsible for the health benefits of certain foods and supplements is the provision of natural antioxidants that may balance age-related oxidative damage and limit the pathological changes associated with neurodegeneration [15,16,17].

**Figure 1:**
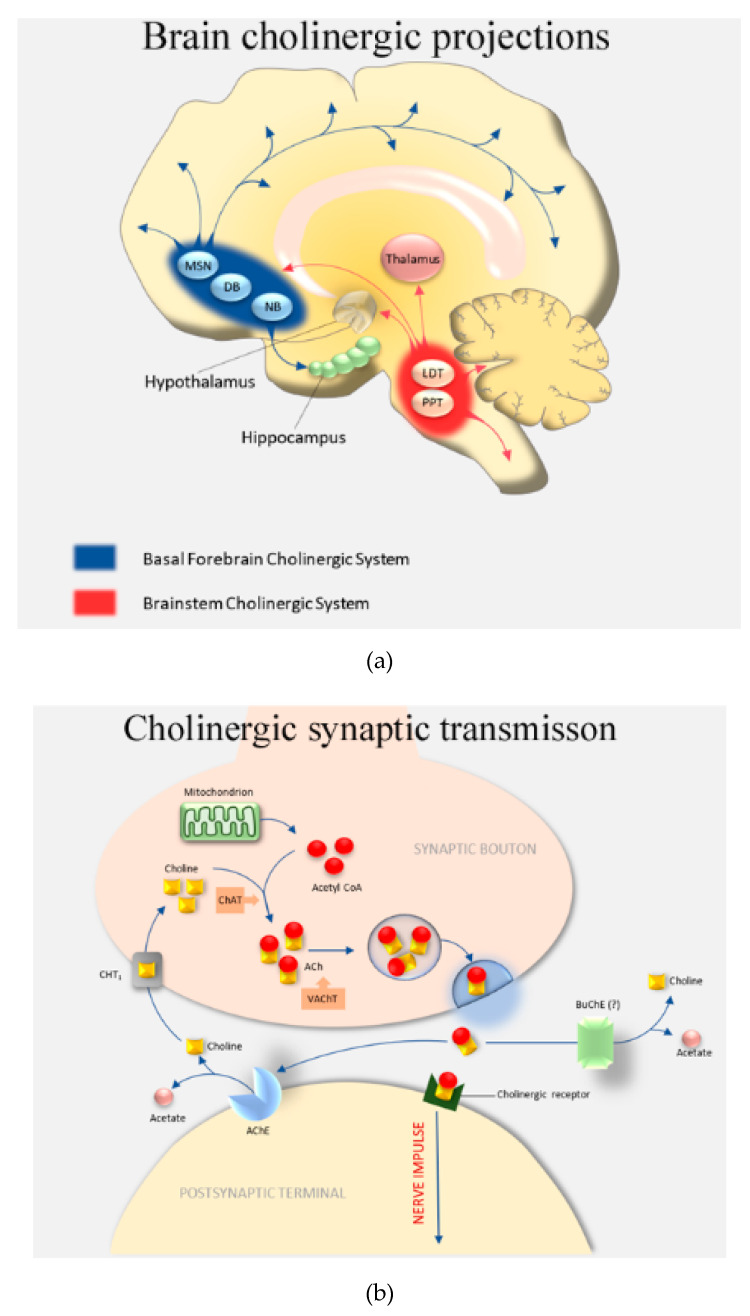
The cholinergic system and cholinergic synapse. (**a**) Overview of the cholinergic system: DB, diagonal band of Broca; LDT, laterodorsal pontine tegmentum; MSN, medial septal nucleus; NB, nucleus basalis; PPT, pedunculopontine tegmental nucleus. (**b**) The cholinergic synapse: ACh, acetylcholine; AChE, acetylcholinesterase; BuChE, butyrylcholinesterase; ChAT, choline acetyltransferase; CHT_1_, choline transporter; VAChT, vesicular acetylcholine transporter.

The neuropathic characterization of the major neurodegenerative disease, AD, is via the deposition of extracellular amyloid plaques of aggregated Aβ, and intracellular neurofibrillary tangles composed of the microtubule-associated protein (MAP) tau, which becomes extensively post-translationally modified (hyperphosphorylated) and aggregated within protein deposits [18,19]. These peptide (Aβ) and protein (tau) aggregates are thought to be toxic and contribute to the loss of neuronal number and cognitive ability, most notably within the frontotemporal lobes, including the entorhinal cortex and hippocampal regions of the brain [18,20]. Induction of oxidative stress and neuro-inflammation have also been proposed to contribute to brain damage in AD [21,22]. The cognitive decline experienced by AD patients is exacerbated by reduced functionality of the cholinergic pathways [22,23] (Figure 1a). 

Cholinergic signaling is mediated by the transport of the neurotransmitter, acetylcholine (ACh), across the synaptic cleft to initiate post-synaptic transmission at nerves or at the neuromuscular junction. Acetylcholinesterase (AChE) is tethered to the post-synaptic membrane and is able to rapidly cleave ACh into acetate and choline and, thereby, terminate ACh stimulation (Figure 1b). The ability to sustain ACh levels through inhibition of AChE is the current mainstay of treatment for mild to moderate AD and is the primary mode of action for the FDA-approved drugs: donepezil, rivastigmine, and galantamine [24,25].

Emerging scientific evidence supports the potential use of dual (acetyl- and butyryl-) cholinesterase inhibitors (ChEIs) [26]. Indeed, the efficacy of rivastigmine may, in part, relate to its dual inhibitor ability [24]. The discovery of plant alkaloids as the source of the ChEIs, rivastigmine and galantamine, has prompted an ongoing search for phytochemicals with anti-cholinesterase activities. Phytochemicals and plant extracts may also possess a range of secondary metabolites with antioxidant activity and low target cell toxicity.

To this end, we investigated the nutraceutical potential of leaves from seven commonly cultivated plants: Lemon (*Citrus limon*), Red silk-cotton (*Bombax ceiba*), Henna (*Lawsonia inermis*), Eucalyptus (*Eucalyptus globulus*), Basil (*Ocimum basilicum*), Mandarin Orange (*Citrus reticulata*), and Spearmint (*Mentha spicata*). These plants were chosen on the basis of their availability, use within folk medicine (including within Pakistan), and previously reported phytochemicals with potential anti-cholinergic activity (Supplementary Table S1).

*Citrus limon* (family Rutaceae) is a small, evergreen tree native to South Asia and grown in regions with temperate summers. The tree is harvested for production of lemons (edible fruit) and lemon juice (harnessed for use in the drinks industry), as well as production of essential oils from lemon peel [27]. Plant leaves are also utilized and extoled for their health benefits when consumed as a tea [28].

*Bombax ceiba* (family Bombacaceae) is a deciduous tree located within tropical and subtropical Asia. The flowers and flower buds are used in cooking and the production of tea, and other tree parts including roots and leaves have been utilized in traditional medicines [29,30].

*Lawsonia inermis* (family Lythraceae) more commonly known as Henna, is a branched, glaborous shrub or small tree commonly located within Africa, Asia, and Australia. Henna leaves contain a broad number of phytochemicals, including lawsome, and have been used historically for temporary skin staining and as a hair dye. Henna has been cultivated for its leaves, flowers, seeds, stem bark, and roots, from which extractions have been used in traditional medicine to treat a broad number of conditions [31,32,33,34].

*Eucalyptus globulus* (family Myrtaceae) is a member of the expansive Eucalyptus genus, native to Australia, and usually distributed within tropical and subtropical regions. Extracts and essential oils from the leaves of Eucalyptus plants including *E. globulus* have been exploited as a traditional medicine for their beneficial pharmacological activities [35,36]. 

*Ocimum basilicum* (family Lamiaceae) is native to Southeast Asia and Africa. It is extensively cultivated for its leaves, utilized as a culinary herb, and essential oil. Plant seeds and flower buds can also be harvested for food or added to drinks. *O. basilicum* (basil) has been utilized for traditional medicines with plant leaves and seeds rich in polyphenols, such as phenolic acids, with antioxidant potential [37,38].

*Citrus reticulata* (family Rutaceae) is another member of the Citrus genus and is believed to be native to Southeast Asia. *C. reticulata* is grown and harvested for its edible, soft, citrus fruit and peel utilized for essential oil and traditional medicines [27]. 

*Mentha spicata* (family Lamiaceae) is a gastronomic herb native to Europe and Southern Asia, with leaves cultivated for use in foods and teas, and utilized as a flavoring and aromatic oil. Plant extracts have also been traditionally utilized for their medicinal properties throughout the Mediterranean [39].

## 2. Materials and Methods 

### 2.1. Plant Samples and Chemicals

Acetylcholinesterase (AChE) (EC 3.1.1.7), butyrylcholinesterase (BuChE) (EC 3.1.1.8), acetylthiocholine iodide (ATCI), 5,5′-dithiobis-(2-nitrobenzoic acid) (DTNB), galantamine hydrobromide (C_17_H_21_NO_3_.HBr; 368.27 g/mol), 2,2-diphenyl-1-picrylhydrazyl (DPPH), α-tocopherol (Vitamin E), thiazolyl blue tetrazolium bromide (MTT), isopropanol, and dimethyl sulfoxide (DMSO) were purchased from Sigma Aldrich (Poole, UK). Dulbecco’s Modified Eagle’s Medium (DMEM), fetal bovine serum albumin (FBS), and poly-d-lysine (PDL) were purchased from Gibco (Loughborough, UK). Seven plants of purported medicinal benefit were collected from the Quaid-e-Azam Campus, University of the Punjab, Lahore, and further processed according to botanical standards. Voucher specimens were deposited in the Herbarium of the Botany Department, Abdul Wali Khan University, Mardan, Pakistan, to obtain their respective voucher numbers. The plant leaves studied were from *Citrus limon (AWKUM.Bot.132.3.6.p21)*, *Bombax ceiba (AWKUM.Bot.119.1.1.p1)*, *Lawsonia inermis (AWKUM.Bot.78.3.1.p5)*, *Eucalyptus globulus (AWKUM.Bot.FOC.1.18.p323)*, *Ocimum basilicum (AWKUM.Bot.192.59.2.p294)*, *Citrus reticulata (AWKUM.Bot.132.3.4.p25)*, and *Mentha spicata (AWKUM.Bot.192.48.6.p262)*. 

The phenological stages of the plants were as follows: *Citrus limon*, fruit development; *Bombax ceiba*, senescence; *Lawsonia inermis*, fruit development; *Eucalyptus globulus*, fruit development; *Ocimum basilicum*, flowering; *Citrus reticulata*, fruit development; and *Mentha spicata*, flowering.

### 2.2. Preparation of Plant Extracts

Plant leaves were air-dried and ground to a fine powder using an electric grinder (Cambridge Food Processor, FP 235, Cambridge Electric Company, Cambridge, UK). Powdered plant material (50 g) was wrapped in Whatman filter paper and fixed in the extractor glass tube of a Soxhelt apparatus. Methanol (500 mL) was used as the extraction solvent and poured into the round-bottomed flask of the apparatus. The apparatus was run at 60 °C, with running water used as cooling agent for the condenser. The plant material-to-solvent ratio was kept as 1:10 (*w*/*v*). Extraction was run for five cycles for a total of 4 h. Crude extract collected within the round-bottomed flask at the end of extraction was cooled to room temperature and sieved through Whatman filter paper by funneling into a conical flask. The volume of methanol solvent was reduced using a Rotary Evaporator (4000 Efficient, Heidolph Instruments, Schwabach, Germany). The extracts were then freeze-dried (Alpha 2–4 LD Lyophilizer, Martin Christ, Osterode am Harz, Germany) after adding 50% celite (*w*/*w*) (Sigma-Aldrich, Poole, UK) to generate a fine powder. The extracted (lyophilized) compounds were used for experimentation. 

### 2.3. AChE and BuChE Inhibitory Activity

Measurements of inhibition of AChE or BuChE were determined by a modified Ellman’s procedure [40], adapted for a 96-well plate. Within each well, 40 µL of 0.01 M DTNB, 46 µL of 0.1 M (pH 8.0) phosphate buffer, 2 µL of 0.075 M substrate, and 10 µL of sample extract were mixed. The enzymatic reaction was initiated by the addition of 2 µL of 0.5 U/mL AChE or BuChE and then monitored at 412 nm using a spectrophotometer (Multiskan Spectrum, Thermo Electron Corporation, Vantaa, Finland). Plant extracts were assayed across a concentration range of 0.16, 0.31, 0.63, 1.25, and 2.5 mg/mL, and the percentage of inhibition of AChE or BuChE was calculated relative to no inhibitor controls. All assays were performed in duplicates, with negative controls (no enzyme) values subtracted from all data points. Galantamine was used as a positive control for inhibition of either AChE or BuChE. The concentration of plant extract that inhibited 50% of the cholinesterase activity (IC_50_) was calculated by nonlinear regression using GraphPad Prism V.7 (San Diego, CA, USA; https://www.graphpad.com/scientific-software/prism/). 

### 2.4. Antioxidant Activity 

Antioxidant activity of the plant extracts was determined using a 2,2-diphenyl-1-picryl-hydrazyl-hydrate (DPPH) free radical scavenging assay. Plant extracts, over a concentration range of 12.5, 25, 50, 100, and 200 µg/mL, were prepared in 20 µL of methanol and mixed with 180 µL of 0.1 mM DPPH in 80% (*v*/*v*) methanol in a 96-well microtiter plate. The plate was incubated at 37 °C for 40 min and then optical density of the solution was measured at 517 nm using a spectrophotometer (Multiskan Spectrum, Thermo Electron Corporation), as described in a previous publication [41]. The percentage DPPH radical scavenging (antioxidant) activity was calculated relative to α-tocopherol (vitamin E) used as a positive control. All data points were performed in duplicates, with the absorbance values from negative controls subtracted.

### 2.5. Cytotoxicity Assays

Human neuroblastoma (SH-SY5Y) cells were purchased from the European Collection of Authenticated Cell Culture (ECACC) (ECACC-94030304). SH-SY5Y cells were grown in a culture medium of 43.5% Eagle’s Minimum Essential Medium (EMEM) (M4655, Sigma, Poole, UK) supplemented with 43.5% Ham’s F12 nut mix (217665-029, Gibco, Waltham, MA, USA), 10% heat-inactivated fetal bovine serum (FBS) (F9665, Sigma), 1% MEM Non-Essential Amino Acid Solution (RNBF3937, Sigma), 2 mM glutamine, and 1% penicillin-streptomycin solution containing 10,000 IU penicillium and 10 mg/mL streptomycin (P4333, Sigma) in T_25_ flasks (130189, Thermofisher Scientific, Rochester, UK) at 37 °C within an atmosphere of 5% CO_2_ and 95% humidity. SH-SY5Y cells (passage 15) were seeded in 96-well plates at 3 × 10^4^ cells/well and grown until 80–90% confluence before treatment with plant extracts at 156, 312, 625, 1250, or 2500 µg/mL. After 48 h, cell culture media was removed and replaced with 0.5% (*w*/*v*) thiazolyl blue tetrazolium bromide (MTT) reagent containing growth media. Plates were incubated at 37 °C for 4 h before removal of media and replacement with 1:1 (*v*/*v*) isopropanol:DMSO, and absorbance was recorded at 570 nm using a spectrophotometer (Multiskan Spectrum, Thermo Electron Corporation). The effect of different concentrations of plant extracts was determined by comparing the viability of treated cells with untreated control cells, and the plant concentration able to inhibit cell viability by 50% (IC_50_) was calculated by nonlinear regression using GraphPad Prism V.7.

### 2.6. Liquid-Chromatography Mass-Spectrometry (LC-MS)

Plant extracts were analyzed using reversed-phase (Phenomenex; Jupiter C18; 4.6 × 250mm, 5 µm 300 Å) (Agilent, Cheshire, UK) high-pressure liquid chromatography with a 5–60% (*v*/*v*) gradient of acetonitrile in 0.1% (*v*/*v*) formic acid chromatographed at 1.0 mL.min^−1^, with 90% of flow directed to a microplate fraction collector (Dionex; Foxy Jr, Themofisher, Loughborough, UK). Mass spectrometry (QToF Premier, Waters, UK) was performed in negative ion electrospray mode, with instrument control and externally calibrated (sodium iodide; similar to 10 ppm), and data processed using MassLynx 4.1 software (Waters, UK).

### 2.7. In Silico Molecular Docking Studies

The 3D structures of human AChE (PDB ID: 4EY5) and BuChE (PDB ID: 6I0C) enzymes were downloaded from RCSB-Protein Data Bank (PDB) (https://www.rcsb.org/). The 3D structures of the potential ChEIs identified by LC-MS/MS were downloaded from the National Center for Biotechnology Information PubChem database (https://pubchem.ncbi.nlm.nih.gov/). The 3D structures of these potential ligands were docked with the target enzymes AChE and BuChE separately using AutoDock Vina software and MGL tools (The Scripps Research Institute, La Jolla, CA, USA) [42]. Molecular docking results were visually analyzed using PyMOL v2.4. (Schrödinger) and the ligands ranked with regards to their binding affinities to the target enzymes.

### 2.8. Statistical Analysis

Statistical analysis was performed using GraphPad Prism V.7. Determination of IC_50_ values were obtained by nonlinear regression (inhibitor vs. normalized response-variable slope) based on a best-fit model from at least four data points. Comparison between groups was performed using a one-way analysis of variance (ANOVA). A Spearman rank-order correlation coefficient was used to quantify the relationship between the studied parameters. A *p* value of below 0.05 was considered significant.

## 3. Results

### 3.1. Assessment of Plant Extract AChE and BuChE Inhibitory Activity

**Figure 2:**
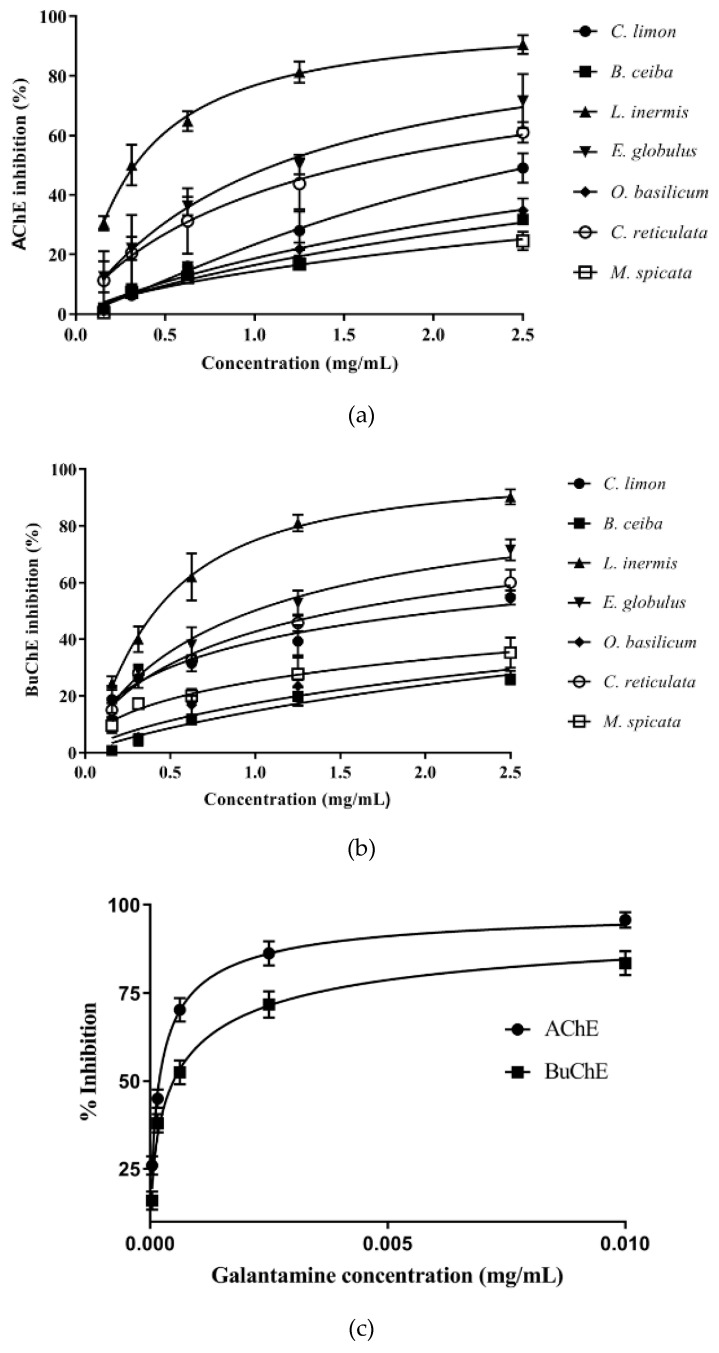
Assessment of the ability of plant extracts and galantamine to inhibit AChE and BuChE. (**a**) Inhibition of AChE by plant extracts. (**b**) Inhibition of BuChE by plant extracts. (**c**) Inhibition of AChE and BuChE by galantamine. Assays were performed using modified Ellman’s assay [40]. Results are expressed as means ± SEM, for an *n*-number of 4.

Plant extracts were scrutinized across a concentration range of 0.16–2.5 mg/mL for their ability to inhibit AChE or BuChE using a modified Ellman’s spectrophotometric assay. Plant extracts inhibited the cholinesterases in a concentration-dependent manner (Figure 2a,b). The actual or extrapolated concentration able to produce 50% inhibition (IC_50_) of AChE or BuChE was calculated by nonlinear regression (Table 1). The anti-cholinesterase drug, galantamine, was used to provide a comparison of AChE and BuChE inhibitory activity. Galantamine inhibitor-response curves for AChE and BuChE are included in Figure 2c. From nonlinear regression, IC_50_ values for galantamine vs. AChE and BuChE were calculated as 0.19 µg/mL (0.51 µM) and 0.49 µg/mL (1.3 µM), respectively (Supplementary Figure S1), and are included in Table 1. 

**Figure 3:**
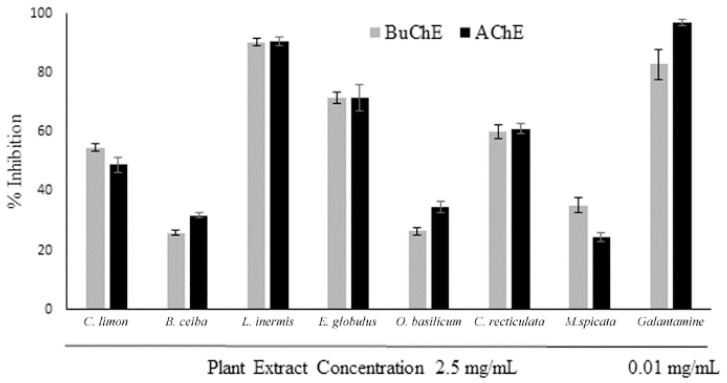
Comparison of BuChE and AChE inhibitory activity for plant extracts. The ability of plant extracts at 2.5 mg/mL to inhibit BuChE and AChE was compared with that for galantamine at 0.01 mg/mL. Results are expressed as means ± SEM, for an *n*-number of 4.

Henna (*Lawsonia inermis)* leaf extract was the most potent plant inhibitor of both AChE and BuChE, with IC_50_ values of 0.33 and 0.41 mg/mL, respectively (Table 1). Interestingly, the majority of these plant extracts were equipotent inhibitors of AChE and BuChE (Figure 3 and Table 1). Galantamine inhibited AChE and BuChE close to maximal levels, as did the methanolic extract of *L. inermis* (Figure 3). AChE inhibitory potency for the plant extracts was in the order *L. inermis* > *E. globulus* > *C. reticulata* > *C. limon* > *O. basilicum* > *B. ceiba* > *M. spicata*. BuChE inhibitory potency was similar in order for the first four extracts: *L. inermis* > *E. globulus* > *C. reticulata* > *C. limon* > *M. spicata* > *O. basilicum* > *B. ceiba* (Figure 3 and Table 1).

### 3.2. Assessment of Plant Extract Antioxidant Activity

**Figure 4:**
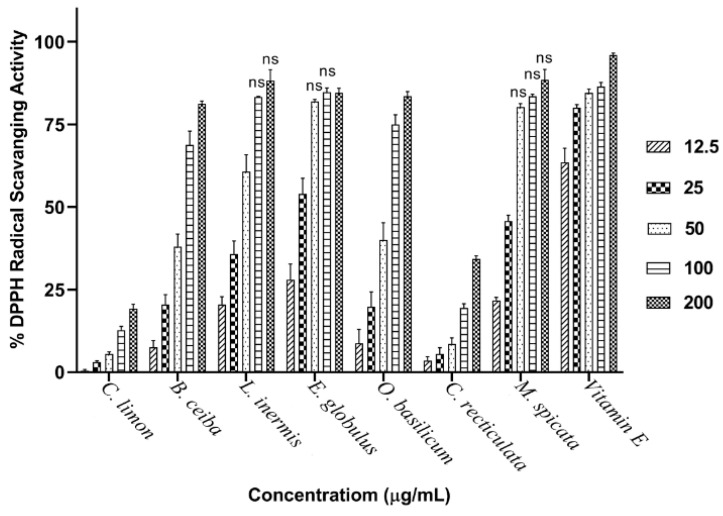
DPPH radical scavenging activity of plant extracts. The ability of plant extracts across a concentration range of 12.5–200 µg/mL to scavenge the DPPH^•^ was assessed spectrophotometrically and compared with Vitamin E. Histograms represent means ± SEM for an n-number of 4; ns = nonsignificant differences from Vitamin E.

Intrinsic antioxidant activity for the plant extracts was assessed via the ability to scavenge free radicals using a DPPH assay. All plant extracts displayed free radical scavenging (antioxidant) ability that was extract concentration dependent (Figure 4). The antioxidant capacity of the plant extracts was *E. globulus* > *M. spicata* > *L. inermis* > *O. basilicum > B. ceiba*, generating similar hyperbolic curves (Supplementary Figure S2). By comparison, the antioxidant capability was relatively low for the citrus fruits, *C. limon* and *C. reticulata*, and increased linearly with extract concentration (Supplementary Figure S2). At the higher concentrations examined (50–200 µg/mL), radical scavenging was to a similar level as the antioxidant vitamin E for several of the extracts (Figure 4). The concentration of extract able to produce a 50% effect (ED_50_) was determined by nonlinear regression (Supplementary Figure S1), with values included in Table 1. 

### 3.3. Assessment of Plant Extract Neuronal Toxicity

**Figure 5:**
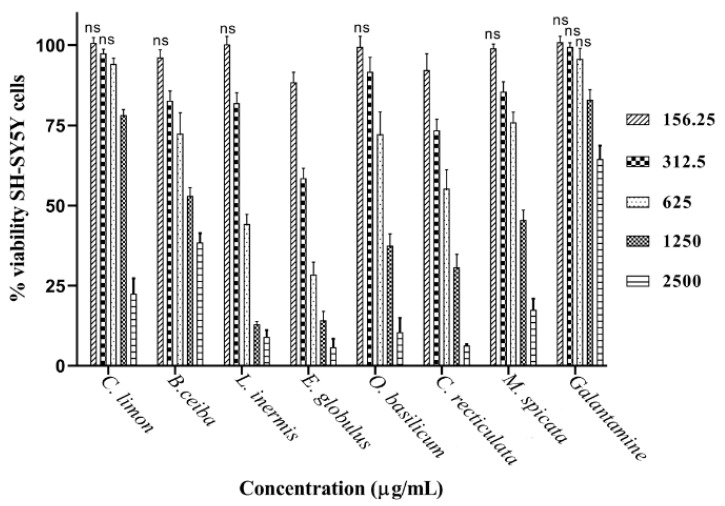
Toxicity of plant extracts to SH-SY5Y neuroblastoma cells. Cultured SH-SY5Y cells were exposed to plant extracts over a concentration range of 156–2500 µg/mL and for 48 h. Toxicity was evaluated by a MTT assay. Histograms represent cell viability as means ± SEM for an n-number of 4; ns = nonsignificant differences from control values.

Plant extracts were applied to cultured neuronal (SH-SY5Y) cells over a concentration range of 0.16–2.5 mg/mL to assess neurotoxicity. Toxicity of plant extracts was concentration dependent (Figure 5). At the lowest concentrations applied to cells (0.16 mg/mL) the majority of plant extracts were nontoxic, with no significant reduction in cell viability (Figure 5). From inhibitor-response curves of cell viability (Supplementary Figure S3), IC_50_ concentrations were calculated (Table 1). Toxicity to neuronal cells was in the order *E. globulus > L. inermis* > *C. reticulata* > *O. basilicum > M. spicata > B. ceiba* > *C. limon*. Eucalyptus plant extract was the most toxic (lowest IC_50_) and reduced cell viability by 11.5% at the lowest concentration examined (0.16 mg/mL). In contrast, the pure drug, galantamine, only induced significant toxicity to neuronal cells (reduced viability of 17%) from a concentration of 1.25 mg/mL. The inhibitor-response curve for galantamine toxicity approached 50% at the highest concentration examined (2.5 mg/mL), with a predicted IC_50_ of 3.53 mg/mL (12.2 mM) (refer to Table 1). 

### 3.4. Assessment of Associations between Cholinesterase Inhibition, Radical Scavenging, and Cell Toxicity

**Figure 6:**
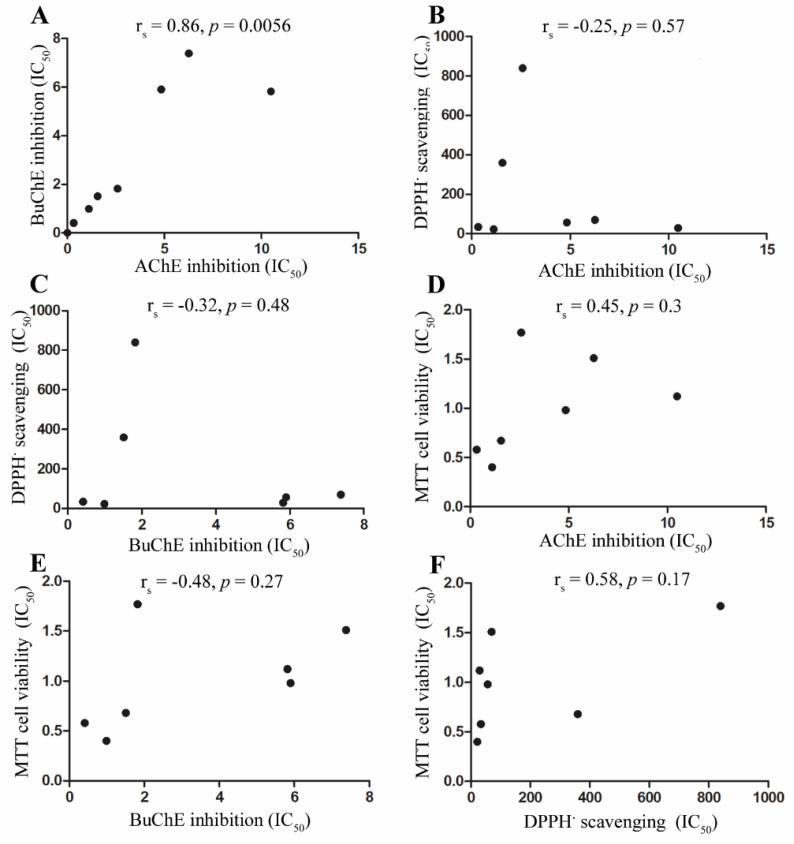
Correlation analysis between anti-cholinesterase activities, DPPH radical scavenging, and cell viability for plant extracts. (**A**) Correlation between BuChE and AChE potency. (**B**) Correlation between DPPH radical scavenging and AChE inhibition. (**C**) Correlation between DPPH radical scavenging and BuChE inhibition. (**D**) Correlation between cell viability and AChE inhibition. (**E**) Correlation between cell viability and BuChE inhibition. (**F**) Correlation between cell viability and DPPH radical scavenging.

To establish if there were any correlations between the studied parameters, a Spearman rank-order correlation coefficient analysis was performed to examine the relationship between AChE and BuChE inhibitory potency (Figure 6A), DPPH radical scavenging ability and cholinesterase inhibition (Figure 6B,C), cell viability and cholinesterase inhibition (Figure 6D,E), and cell viability and DPPH radical scavenging ability (Figure 6F). There was a positive and significant correlation between potency of plant extracts to inhibit AChE and BuChE (Figure 6A). Radical scavenging ability was negatively correlated with either acetyl- or butyryl-cholinesterase inhibitory potency, although not significantly (Figure 6B,C). Cell viability (measured as MTT assays) was positively correlated with AChE inhibitory potency and negatively correlated with BuChE inhibitory potency (Figure 6D,E). Cell viability was positively correlated with radical scavenging potency, but not significantly (Figure 6F). 

### 3.5. Liquid-Chromatography Mass-Spectrometry (LC-MS)

Chemical analyses of the plant extracts were performed using LC-MS/MS, using separation conditions optimized for *Moringa oleifera* [43,44]. This revealed several molecular masses present within all plant extracts, such as rutin (quercetin-3-*O*-rutinoside) and kaempferol 3-*O*-glucoside, as well as others, including 3-caffeoylquinic acid, only present within the two most powerful cholinesterase-inhibiting plant extracts: *L. inermis* and *E. globulus* (refer to Table 2). 

### 3.6. Molecular Docking of Potential ChEIs 

The cleaned 3D structures of AChE (PDB ID: 4EY5) and BuChE (PDB ID: 6I0C) were used as virtual targets for binding by the identified phytochemicals: methyl 4-caffeoylquinate (peak #2), 3-caffeoylquinic acid (peak #9), rutin (peak #13), quercetin 3-*O*-glucoside (peak #14), quercetin-acetyl-glycoside (peak #15), kaempferol 3-*O*-glucoside (peak #16), and kaempferol-acetyl-glycoside (peak #17) (refer to Table 2). The potential ChEIs were ranked according to their respective binding affinities to AChE and BuChE. The 3-caffeoylquinic acid displayed the strongest binding affinity for AChE, and rutin the strongest for BuChE, with binding energies of −9.2 and −11.0 kcal/mol, respectively (Table 3). All these phytochemicals displayed relatively strong binding affinities to AChE and BuChE compared to the commercial ChEI, galantamine (Table 3). Indeed, galantamine was only superior to one of the phytochemicals, quercetin 3-*O*-glucoside, for binding strength to AChE and to one other phytochemical, 3-caffeoylquinic acid, for binding to BuChE (Table 3).

**Figure 7:**
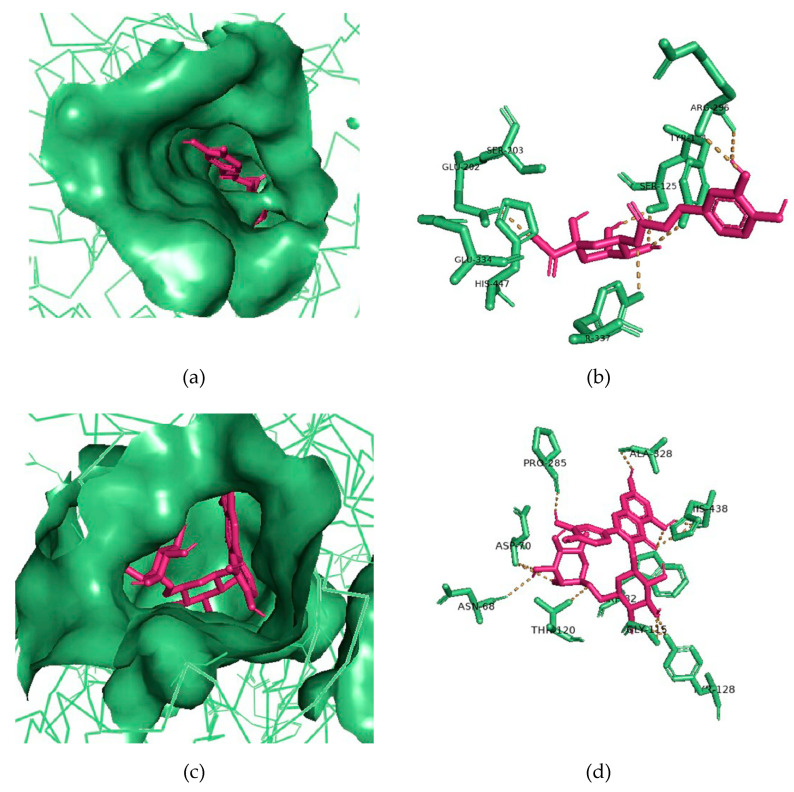
Molecular docking of potential ChEIs. (**a**) Binding pose of 3-caffeoylquinic acid (pink-colored sticks) looking down the gorge of AChE (green-colored surface representation). (**b**) The 3-caffeoylquinic acid (pink-colored sticks) docked with binding site residues of AChE (green-colored sticks). (**c**) Binding pose of rutin (pink-colored sticks) looking down the gorge of BuChE (green-colored surface representation). (**d**) Rutin (pink-colored sticks) docked with binding site residues of BuChE (green-colored sticks). Hydrogen bonding represented by yellow lines.

For the most potent putative AChE inhibitor, 3-caffeoylquinic acid, modeling of its binding to AChE was undertaken. Figure 7a represents the binding pose of 3-caffeoylquinic acid looking down the catalytic gorge of AChE. Figure 7b provides a closer inspection of the binding of 3-caffeoylquinic acid with the active site residues of AChE, demonstrating the hydrogen bonding between the ligand and target enzyme.

Similarly, for the most potent putative BuChE inhibitor, rutin, virtual binding looking down the gorge of BuChE enzyme was performed (Figure 7c), as well as docking of rutin with the active site residues of BuChE (Figure 7d).

## 4. Discussion

Neurodegenerative decline, especially dementia, is a social and economic public health pandemic. Patients suffering from AD represent the greatest number of dementia cases, and deployment of ChEIs are the first-line drugs to abate the cholinergic deficit that contributes to impaired cognition [45]. Phytochemicals with ChEI and antioxidant activities may prove beneficial at both treatment and prophylactic levels. Hence, herein, we scrutinized the ability of leaf extracts from commonly cultivated plants for their nutraceutical efficacy as anti-cholinesterases and antioxidants and considered their toxicity to neuronal cells. 

All the plant extracts examined displayed both AChE and BuChE inhibitory ability, but with broad potency: with an efficacy range of 32-fold for inhibition of AChE and 18-fold for BuChE. *L. inermis* (Henna) leaf extract was the most potent AChE and BuChE inhibitor: with IC_50_ values of 0.33 and 0.41 mg/mL, respectively. Henna (also commonly known as Mhendi) leaves have historically been utilized as a stain and dye, but a comprehensive range of pharmacologically active phytochemicals with desirable health benefits have been reported for leaves and other plant parts [31,32,33,34]. This includes the ability to inhibit AChE: An aqueous leaf extract displayed approximately half of the potency of the methanolic extract reported herein (IC_50_ = 0.749 mg/mL) [46] and other methanolic extracts with relatively weak activity have been described, but without a determination of an IC_50_ [47]. Hence, to date, our results appear to be the first determination of an IC_50_ for a methanolic extract from Henna. Furthermore, this is the first report of BuChE inhibitory activity from Henna leaf extract and, moreover, with comparable inhibitory potency to that against AChE.

The biological roles of BuChE have not been fully delineated. Serum BuChE activity has functional effects upon drug detoxification [48]. BuChE activity also influences lipid metabolism and affects appetite through modification of ghrelin [49,50]. BChE-deficient mice do not display signs of ill health and, similarly, humans devoid of active BChE activity have no reduction in longevity [51,52]. Nevertheless, BuChE is able to substitute for AChE, with AChE mice viable [53]. Furthermore, in AD, BuChE levels increase, presumably to compensate for dwindling ACh functionality [23,54,55]. Hence, there may be additional benefit to the development and utilization of dual transient inhibitors of both cholinesterases to prolong cholinergic signaling [26,56]. For Henna, it is noteworthy that nootropic benefits have already been reported in vivo [57]. So, further development of Henna extracts could provide suitable AD-modifying agents.

For the Henna methanolic leaf extract, we also confirmed the presence of antioxidants using a free radical scavenging spectrophotometric assay. This technique employs the commercially available 1,1-diphenyl-2-picrylhydrazyl free radical (DPPH^•^), which displays a characterized UV-visible spectrum with absorbance maxima at approximately 515 nm in methanol. The provision of an antioxidant, such as a polyphenol, results in a decrease of absorbance that is proportional to the concentration and antioxidant activity of the compound itself [58,59]. In keeping with our results, other independent studies have similarly highlighted the presence of a range of Henna phytochemicals able to act as antioxidants [46,60].

*E. globulus* (Eucalyptus) was the next most potent inhibitor of both cholinesterases. Specific reports of anti-AChE or anti-BuChE activities from extracts of *E. globulus* are absent in the literature. However, anti-AChE activity has been demonstrated for *E. globulus* essential oil both in vitro [61] and in vivo [62]. Essential oils differ from the solvent extracts utilized for our study, since they are generated primarily through hydrodistillation and via steam or dry distillation processes. Nevertheless, it is pertinent to consider that certain essential oils are a source of agents with potential beneficial effects on cognition [63], responses that could, in part, reflect phytochemicals capable of modifying cholinesterase activities. 

For the plants examined, *E. globulus* displayed the best antioxidant potency. This ability to provide useful antioxidant and free radical scavenging was also observed for a water extract from *E. globulus* leaves, with an IC_50_ for DPPH of 12 µg/mL, in keeping with our data [64]. This water extract was also able to scavenge free radicals including those from reactive oxygen species (ROS) and reactive nitrogen species (RNS), properties proposed to align with the plant’s therapeutic activities [64]. Notable antioxidant properties from Eucalyptus leaf extracts (as a food additive) [65] and essential oils [66] have also been reported.

The two citrus fruits, *C. reticulata* and then C. *limon*, were the next most powerful inhibitors of both AChE and BuChE. To our knowledge, our results constitute the first report of anti-AChE and anti-BChE activity for *C. reticulata*. Other studies have highlighted potent anti-AChE of *C. limon* [67] that may relate to the abundance of flavonoids within citrus fruit [68,69]. Furthermore, C. *limon* essential oils also contain anti-cholinesterase activities [70] and were recently shown to ameliorate age-associated cognitive decline in a mouse model of AD [71].

In contrast to their useful anti-cholinesterase activities, both citrus fruit extracts were weak antioxidants. Likewise, only low to moderate antioxidant properties have been reported for *C. reticulata* and C. *limon* peel essential oils [61,72]. Nevertheless, irrespective of their relatively weak antioxidant activity, the multitude of phytochemicals from citrus fruits may contribute to their purported neuroprotective properties [73].

*O. basilicum* displayed intermediate anti-cholinesterase activity, as well as antioxidant potential. Our results for anti-AChE potency of a methanolic leaf extract compare well with those from Farag et al. (2006) [74], with an IC_50_ calculated at 6.6 mg/mL. To date, we have not encountered any other published reports of anti-BuChE activity from *O. basilicum*. The antioxidant properties of basil are thought to arise, at least in part, from a rich blend of polyphenolic compounds including phenolic acids and flavonoids [37,38,75,76]. These *O. basilicum* phytochemicals may also contribute to memory enhancement and anti-cholinesterase activity [77,78].

A methanolic leaf extract from *B. ceiba* displayed relatively weak anti-AChE activity, was the weakest anti-BuChE inhibitor, and had intermediate antioxidant potential. Similarly, a methanolic extract from *B. ceiba* flowers had comparable radical scavenging to our data (EC_50_ = 87 µg/mL) [79]. Other solvent extracts (ethanolic and hexane) from *B. ceiba* flowers also retain useful anti-AChE inhibition and free radical scavenging abilities [80]. These *B. ceiba* flower extracts and a methanolic leaf extract have also displayed neuroprotective potential in vivo [81,82]. 

Our results show that *M. spicata* was a strong antioxidant but only a weak cholinesterase inhibitor, although potent AChE inhibition by *Mentha* essential oils, including *M. spicata* (IC_50_ = 0.088 mg/mL), has been demonstrated [83]. In keeping with our results, *M. spicata*’s potency as an antioxidant was also demonstrated for a water and, to a lesser extent, an ethanolic extract (IC_50_ of 5.7 and 65.2 µg/mL, respectively), with both extracts only exhibiting moderate anti-AChE activity [84]. 

This inverse relationship between DPPH radical scavenging and ability to inhibit cholinesterases exhibited by *M. spicata* extracts was also confirmed as a collective for the seven plant extracts (Figure 6B,C), such that stronger antioxidant potential correlated with weaker cholinesterase inhibition, although this did not reach significance.

The toxicity of the plant extracts to neuroblastoma cells was examined using the universally utilized MTT assay. This assay relies upon the reduction of MTT to formazan by non-mitochondrial and mitochondrial oxidoreductase enzymes. The color change produced directly correlates with cellular metabolic activity and thereby acts as a surrogate of cell viability or proliferation [85,86] and generates inhibitor-response curves that invariably follow those for other markers of cell viability such as ATP depletion or lactate dehydrogenase (LDH) release [87,88]. 

The inhibitor-response curves for the plant extracts revealed a toxicity range of 4.4-fold from the least toxic, *Citrus limon*, to the most toxic, *Eucalyptus globulus*, with all extracts more toxic than the single drug treatment of galantamine. Nevertheless, although such partially pure extracts displayed toxicity to neuronal cells, this was at concentrations significantly higher than that for radical scavenging. Indeed, functional antioxidant activity was at concentrations at least one order of magnitude below those that induced toxicity, except for the citrus fruit extracts (*C limon* and *C. recticulata*). In contrast, anti-cholinesterase activity of the plant extracts was at concentrations more comparable to those able to induce neuronal toxicity. The exception was the Henna extract that not only contained the most potent anti-cholinesterases but was also an active inhibitor at concentrations below those able to induce toxicity.

Cellular toxicity and cell death can be induced as a consequence of irreversible inhibition of acetyl-cholinesterase, such as through organophosphate binding, with induction of redox stress [89,90]. By contrast, the current anti-AD medication elicits less toxicity than organophosphate compounds due to only transient inhibition of cholinesterases [24,25]. Our correlation analyses showed that toxicity and loss of cell viability was positively correlated to AChE inhibition, but this did not reach significance (Figure 6D). Cell viability was also negatively correlated with BuChE inhibition, indicating that the most potent BuChE inhibitors may be well tolerated. 

Correlation analysis also demonstrated a significant positive association between AChE inhibition and BuChE inhibition. Thus, phytochemicals present in the plant extracts could potentially include desirable dual cholinesterase inhibitors [26] or mixtures of anti-AChE and anti-BuChE agents. It is also possible that these different plants contain a number of anti-cholinesterases or the same agent(s) but at differing concentrations. 

Lastly, correlation analysis between cell viability and radical scavenging demonstrated a positive relationship, although this did not reach significance (Figure 6F). A positive correlation suggests that, at least for some of the plant extracts, higher (desirable) radical scavenging is associated with more (undesirable) toxicity. Indeed, beneficial antioxidants could suppress elevation of reactive radicals liberated in response to phytochemical redox stress. Certainly, there is a need to be mindful that some polyphenols, including certain flavonoids, are cytotoxic in vitro [91] and, if consumed in excess, may be toxic in vivo [92]. Thus, plant extracts can be double-edged, with a trade-off between provision of both beneficial and refractory polyphenols. 

To investigate further the phytochemical composition of the plant extracts, we performed LC-MS/MS. This revealed several molecular masses that were present in all the plant extracts, such as rutin (quercetin-3-*O*-rutinoside) and kaempferol 3-*O*-glucoside, as well as others, including 3-caffeoylquinic acid, only detected within the two most powerful cholinesterase-inhibiting plant extracts: *L. inermis* and *E. globulus* (refer to Table 2). Consistent with our studies, 3-caffeoylquinic acid was recently demonstrated to exhibit both AChE and BuChE inhibitory capabilities [93,94]. Furthermore, other flavonoids, including rutin, are also capable of inhibiting AChE [95]. Hence, these plant extracts likely retain a range of ChEIs of polyphenol origin. Furthermore, from an in silico molecular modeling approach (Table 3 and Figure 7) [96], an assessment of their potency as ChEIs was undertaken and compared to the current FDA-approved drug, galantamine. However, it is appreciated that these plant extracts and phytochemicals will require further evaluation of their therapeutic potential in vivo.

## 5. Conclusions

In conclusion, we demonstrated that certain commonly harvested plants retain functional and potentially beneficial dual cholinesterase inhibitors as well as substantive antioxidant properties. From further purification and characterization of resident phytochemicals that retain beneficial pharmacology and with reduced adverse drug reactions, suitable agents may be isolated to provide nutraceuticals able to stave off the cognitive decline experienced with age and by AD patients. 

## Figures and Tables

**Table 1:** AChE and BuChE inhibitory capacity, DPPH radical scavenging, and cell viability assessment (MTT assay) of plant extracts.

Agent	Alternative or Common Name	IC_50_ (mg/mL)		ED_50_ (µg/mL)	IC_50_ (mg/mL)
		AChE	BuChE	DPPH	MTT
*Citrus limon*	Lemon	2.59 ± 0.14	1.82 ± 0.10	839.40 ± 135.2	1.77 ± 0.03
*Bombax ceiba*	Red silk-cotton	6.26 ± 0.72	7.38 ± 0.96	69.69 ± 3.01	1.51 ± 0.08
*Lawsonia inermis*	Henna	0.33 ± 0.02	0.41 ± 0.02	33.68 ± 1.04	0.58 ± 0.02
*Eucalyptus globulus*	Eucalyptus	1.11 ± 0.07	0.99 ± 0.05	22.15 ± 1.23	0.40 ± 0.01
*Ocimum basilicum*	Basil	4.84 ± 0.47	5.90 ± 0.81	56.13 ± 2.01	0.98 ± 0.03
*Citrus reticulata*	Mandarin	1.56 ± 0.20	1.51 ± 0.09	359.30 ± 31.64	0.68 ± 0.03
*Mentha spicata*	Spearmint	10.49 ± 2.06	5.82 ± 0.77	28.94 ± 0.96	1.12 ± 0.03
Galantamine	Galanthamine	0.00019	0.00049	ND	3.53 ± 0.21
Vitamin E	α-tocopherol	ND	ND	7.73 ± 0.66	ND

**Table 2:** LC-MS/MS identification of plant phytochemicals.

Peak #	[M-H]	Putative Molecular Formula	Putative Identification	M. oleifera *	*C. lemon*	*B. ceiba*	*L. inermis*	*E. globulus*	*O. basilicum*	*C. reticulata*	*M. spicata*
**1**	665	C_24_H_42_O_21_	Cellotetraose	x	665.17	x	x	x	x	665.17	x
**3**	341	C_12_H_22_O_11_	Sucrose	341.11	341.11	341.11	341.10	341.09	341.11	341.09	x
**4**	503	C_18_H_32_O_16_	Cellotriose	503.15	503.17	503.17	x	503.14	x	x	503.14
**2**	367	C_17_H_20_O_9_	Methyl 4-caffeoylquinate	367.10	367.14	367.16	x	x	x	x	x
**5**	278	C_14_H_17_NO_5_	Niazirin	278.07	x	x	x	x	x	x	x
**6**	191	C_7_H_12_O_6_	Quinic acid isomer 1	191.06	191.06	191.04	x	191.05	x	191.05	x
**10**	191	C_7_H_12_O_6_	Quinic acid isomer 2	191.02	x	191.02	x	x	x	x	x
**7**	586	C_20_H_29_NO_15_S_2_	3-Hydroxy-4-(α-l-rhamnopyranosyloxy) benzyl glucosinolate	586.09	x	x	x	x	x	x	x
**8**	570	C_20_H_29_NO_14_S_2_	Glucomoringin	570.07	x	x	x	x	x	x	x
**11**	408	C_14_H_19_NO_9_S_2_	Glucotropaeolin	x	x	x	x	x	x	x	408.12
**12**	612	C_22_H_31_NO_15_S_2_	Acetyl-4-(α-l-rhamnopyranosyloxy) benzyl glucosinolate	612.07	x	x	x	x	x	x	x
**9**	353	C_16_H_18_O_9_	3-Caffeoylquinic acid	353.08	x	x	353.08	353.07	x	x	353.06
**13**	609	C_27_H_30_O_16_	Rutin (quercetin-3-*O*-rutinoside)	609.14	609.14	609.14	609.14	609.13	609.12	609.14	609.13
**14**	463	C_21_H_20_O_12_	Quercetin 3-*O*-glucoside	463.08	x	463.08	463.09	463.07	463.07	x	x
**15**	505	C_23_H_22_O_13_	Quercetin-acetyl-glycoside	505.13	x	505.26	x	505.11	505.11	505.25	x
**16**	447	C_21_H_20_O_11_	Kaempferol 3-*O*-glucoside	447.08	447.09	447.09	447.07	447.07	447.07	447.06	447.06
**17**	489	C_23_H_22_O_12_	Kaempferol-acetyl-glycoside	489.10	x	x	489.09	489.08	x	489.07	489.13

* *M. oleifera* was used as a reference extract for peak generation according to Xu et al. [44]. x denotes that the compound was not detected in that peak fraction.

**Table 3:** AChE and BuChE binding characteristics of the plant phytochemicals.

Ligand	2D Structures	Binding Affinity (kcal/mol)	
		AChE	BuChE
Galantamine	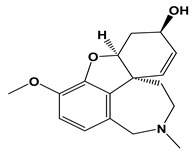	−7.7	−8.7
3-Caffeoylquinic acid	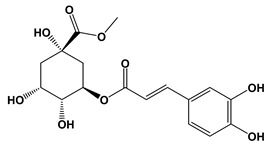	−9.2	−8.6
Methyl 4-caffeoylquinate	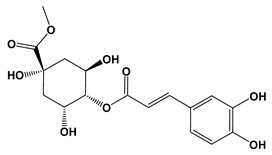	−8.8	−8.9
Kaempferol-acetyl-glycoside	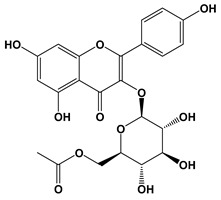	−8.4	−10.4
Quercetin 3-rutinoside (Rutin)	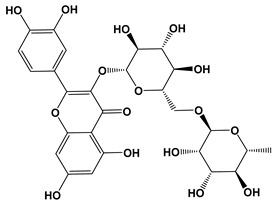	−8.3	−11.0
Quercetin-acetyl-glycoside	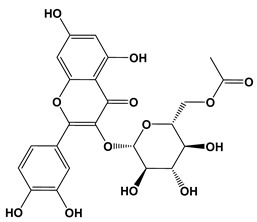	−8.0	−10.4
Kaempferol 3-*O*-glucoside (Astragalin)	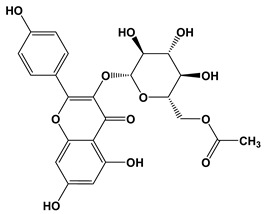	−7.9	−9.7
Quercetin 3-*O*-glucoside (Isoquercitrin)	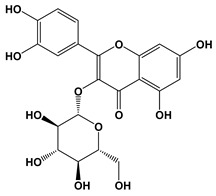	−7.6	−9.5

## Supplementary Materials

The following are available online at https://www.mdpi.com/2218-273X/10/11/1556/s1. Figure S1: Inhibition of AChE and BuChE by galantamine. Figure S2: DPPH radical scavenging activity of plant extracts. Figure S3: Toxicity of plant extracts to neuronal cells. Table S1: Reported phytochemicals with anti-cholinergic activity.
