# Supplementary material for: Potential Nutraceutical Properties of Leaves from Several Commonly Cultivated Plants
Source: Biomolecules. 2020 Nov 15;10(11):1556. doi: 10.3390/biom10111556 (PMC7698063; doi:10.3390/biom10111556)
Supplement: Supplementary file 1 [file biomolecules-10-01556-s001.zip › Supplementary Table S1.pdf]

Supplementary Table S1: Reported phytochemicals with anti-cholinergic activity.

Sr.	Plant Species	Common Name	Example Anticholinergic Compounds
1	*Citrus limon*	Lemon	Limonene, 1,8-cineole, linalool, terpinen-4-ol, α-terpinene, carvone, naringenin, *p*-cymene [1]
2	*Lawsonia inermis*	Henna	Hexane, chloroform, ethanolic and methanolic extracts [2,3]
3	*Eucalyptus globulus*	Eucalyptus	1,8-Cineole, α-terpinene, carvone, limonene, linalool, menthol, naringenin, *p*-cymene, terpinen-4-ol, viridiflorol [1]
4	*Ocimum basilicum*	Basil	1,8-Cineole, α-terpinene, carvone, elemol, limonene, linalool, menthol, menthone, *p*-cymene, terpinen-4-ol [1]
5	*Citrus recticulata*	Mandarin	Naringenin, 1,8-cineole, α-terpinene, carvone, limonene, elemol, linalool, *p*-cymene, terpinen-4-ol [1]
6	*Mentha spicata*	Mint	Menthol, menthone, (+)-piperitenone-oxide, (+)-pulegone, 1,8-cineole, α-terpinene, carvone, elemol, limonene, linalool, *p*-cymene, piperitenone, pulegone, terpinen-4-ol, viridiflorol [1]
7	*Bombax ceiba*	Red silk-cotton	Ethanol, hexane and methanolic extracts [4,5]

References

1. Duke, J.A. *Dr. Duke's Phytochemical and Ethnobotanical Databases*. 2016 (accessed 28/09/2020); Available from: https://phytochem.nal.usda.gov/phytochem/search/list.
2. Rajesh, V., et al., *Memory enhancing activity of Lawsonia inermis Linn. leaves against scopolamine induced memory impairment in Swiss albino mice.* Oriental Pharmacy and Experimental Medicine, 2017. **17**(2): p. 127-142.
3. Chaibi, R., et al., *Assessment of antioxidant, anti-inflammatory, anti-cholinesterase and cytotoxic activities of Henna (Lawsonia inermis) flowers.* J Nat Prod, 2015. **8**: p. 85-92.

4. Sinha, S., et al., *Neuroprotective potential of Cucurbita maxima Duchesne ex Poir, Caeselpenia bunduc (L.) Roxb and Bombax ceiba Linn extracts.* South African journal of botany, 2019. **120**: p. 319-325.
5. Mostafa, N.M., *β-Amyrin rich Bombax ceiba leaf extract with potential neuroprotective activity against scopolamine-induced memory impairment in rats.* Records of Natural Products, 2018. **12**(5): p. 480.
