# Supplementary figures and images for: Potential Nutraceutical Properties of Leaves from Several Commonly Cultivated Plants
Source: Biomolecules. 2020 Nov 15;10(11):1556. doi: 10.3390/biom10111556 (PMC7698063; doi:10.3390/biom10111556)

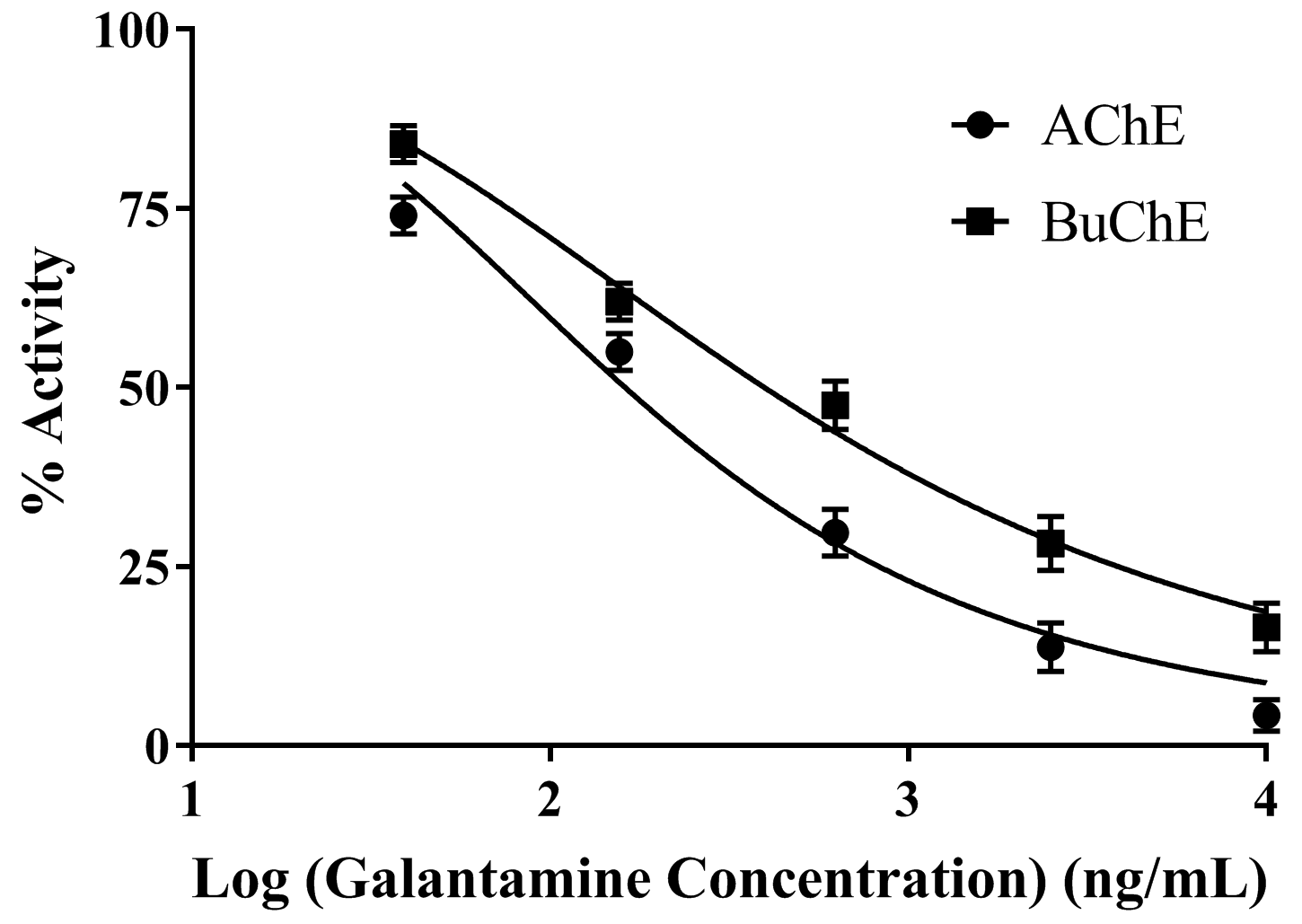

Supplement: Supplementary file 1 [file biomolecules-10-01556-s001.zip › Supplementary Figure S1.tif]

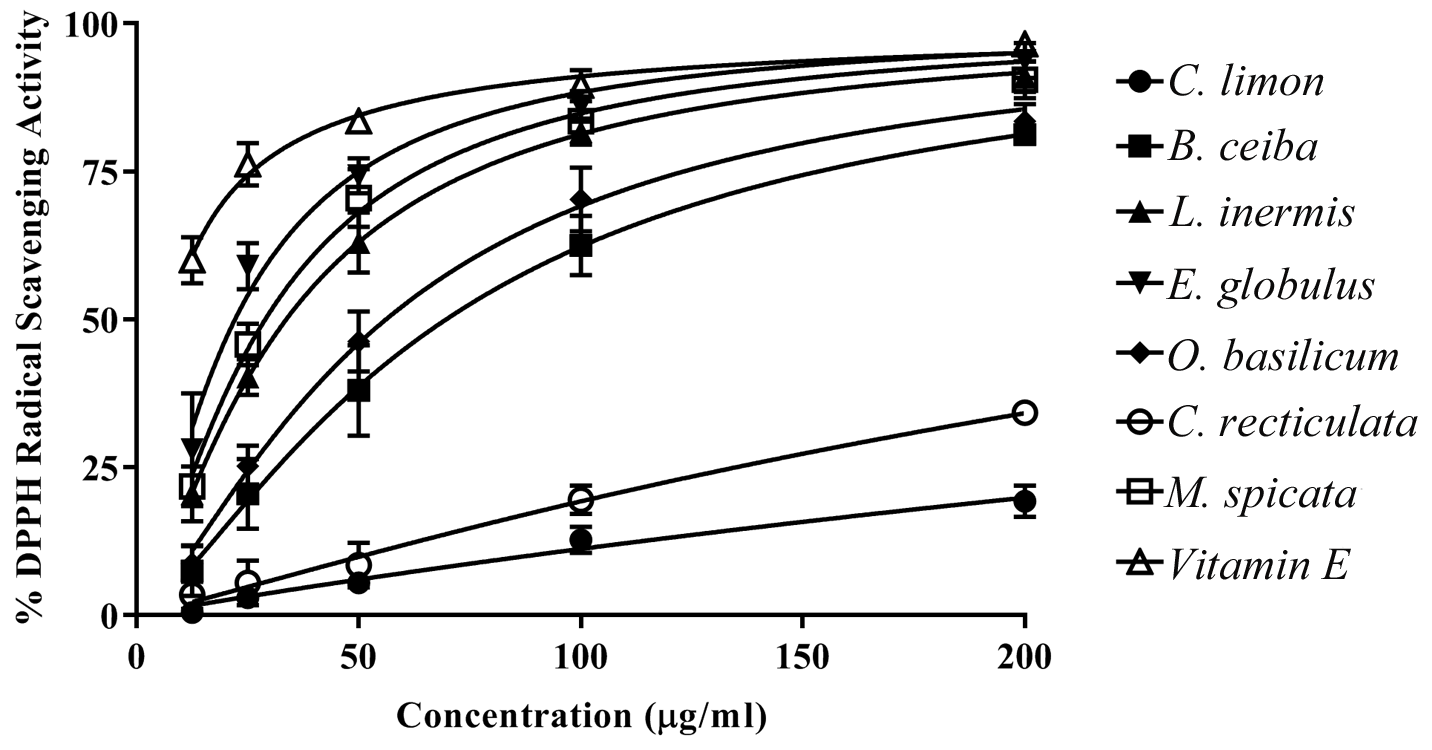

Supplement: Supplementary file 1 [file biomolecules-10-01556-s001.zip › Supplementary Figure S2.tif]

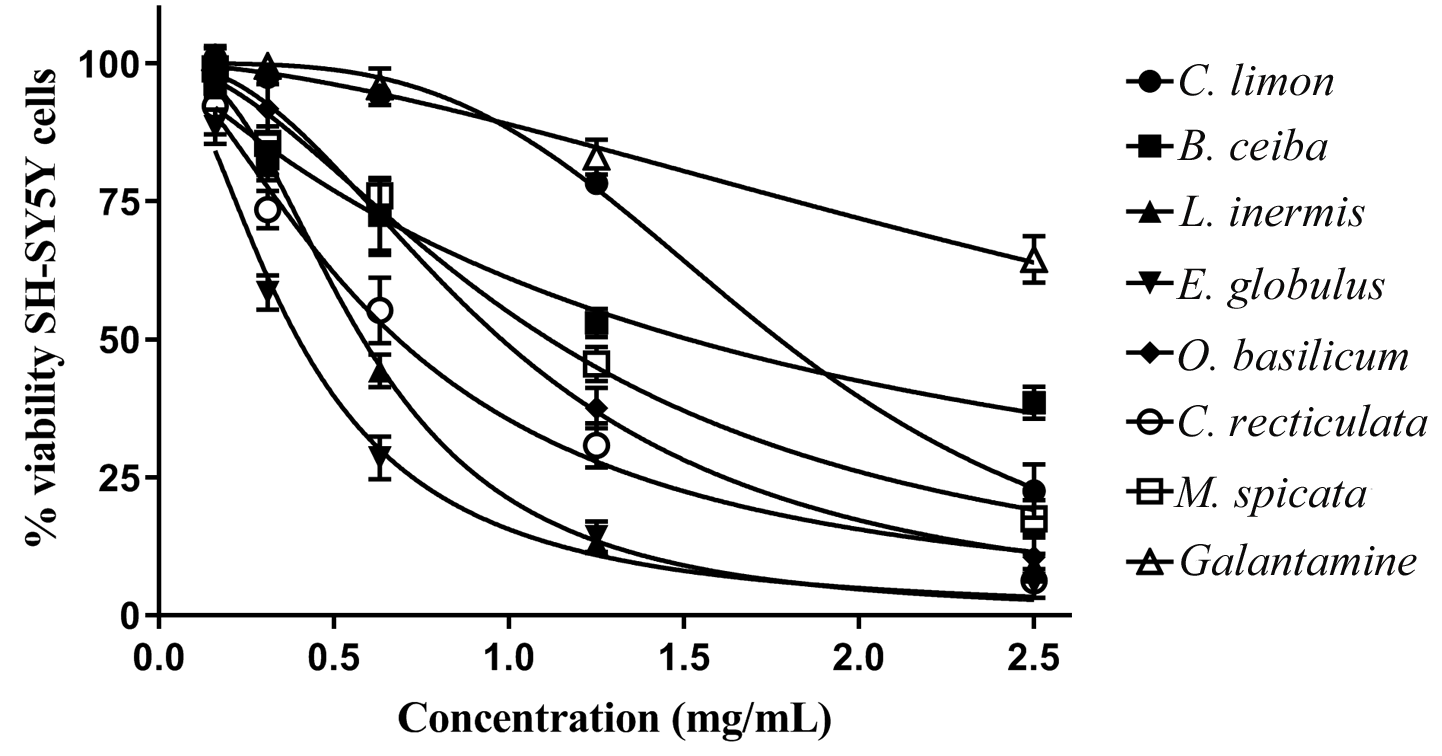

Supplement: Supplementary file 1 [file biomolecules-10-01556-s001.zip › Supplementary Figure S3.tif]
